# Transcriptome and metabolome analyses reveal transcription factors regulating ganoderic acid biosynthesis in *Ganoderma lucidum* development

**DOI:** 10.3389/fmicb.2022.956421

**Published:** 2022-08-04

**Authors:** Li Meng, Ruyue Zhou, Jialong Lin, Xizhe Zang, Qingji Wang, Panmeng Wang, Li Wang, Zhuang Li, Wei Wang

**Affiliations:** Shandong Provincial Key Laboratory of Agricultural Microbiology, College of Plant Protection, Shandong Agricultural University, Tai’an, China

**Keywords:** development, medicinal fungi, regulatory mechanisms, secondary metabolites, *Ganoderma lucidum*

## Abstract

*Ganoderma lucidum* is an important medicinal fungus in Asian countries. Ganoderic acid (GA) is the major variety of bioactive and medicative components in *G. lucidum*. Biosynthesis of secondary metabolites is usually associated with cell differentiation and development. However, the mechanism underlying these phenomena remain unclear. Transcription factors play an essential regulatory role in the signal transduction pathway, owing to the fact that they represent the major link between signal transduction and expression of target genes. In the present study, we performed transcriptome and metabolome analyses to identify transcription factors involved in GA biosynthesis during development of *G. lucidum*. Transcriptome data revealed differentially expressed genes between mycelia and primordia, as well as between mycelia and the fruiting body. Results from gene ontology enrichment analysis and metabolome analyses suggested that GAs and flavonoids biosynthetic process significantly changed during fungal development. The analysis of predicted occurrences of DNA-binding domains revealed a set of 53 potential transcription factor families in *G. lucidum*. Notably, we found homeobox transcription factor and velvet family protein played important role in GA biosynthesis. Combined with previous studies, we provided a model diagram of transcription factors involved in GA biosynthesis during fruiting body formation. Collectively, these results are expected to enhance our understanding into the mechanisms underlying secondary metabolite biosynthesis and development in fungi.

## Introduction

Fungi are remarkable organisms that readily produce a wide range of natural products often called secondary metabolites (Calvo et al., 2002), some of which are beneficial to humankind (Demain and Fang, 2000). *Ganoderma* spp., an important medicinal fungus, has been widely used to improve health and prevent certain diseases in traditional Chinese medicine for thousands of years (Ahmad, 2018), owing to a variety of biomedical efficacies including anti-cancer, anti-tumor, immune modulatory effects. Results from phytochemical studies over the last 40 years have resulted in isolation of 431 secondary metabolites from various *Ganoderma* species. The major secondary compounds isolated from these species include C30 lanostanes, C27 lanostanes, C24, C25 lanostanes, C30 pentacyclic triterpenes, meroterpenoids, and farnesyl hydroquinones, and so on (Baby et al., 2015).

Ganoderic acids (GAs), a triterpenoid produced by *Ganoderma lucidum* that has a range of biological activities, is regulated by interplay between external (environmental) and internal (genetic) factors. Previous studies have shown that expression of GA biosynthetic machinery can be modulated by various external factors, including salicylic acid (Cao et al., 2017), sodium acetate (Meng et al., 2019), and ethylene (Zhang et al., 2017a; Meng et al., 2022), and so on. Additionally, genes encoding 3-hydroxy-3-methylglutaryl CoA reductase (HMGR) (Shang et al., 2008), squalene synthase (SQS) (Zhao et al., 2007), and oxidosqualene cyclase (OSC) (Shang et al., 2010) as well as other important transcription factors reportedly contribute to GA biosynthesis.

Although biosynthesis of natural products has been associated with cell differentiation and development, previously reports have demonstrated that mutants of transcription factors not only affect mycelia morphology, growth rate, hypha branch, primordia and fruiting body formation, but also play a role in biosynthesis of secondary metabolites. Notably, Cary et al. (2017) revealed that homeobox transcription factor gene *hbx1* is required for the development and aflatoxin biosynthesis in *Aspergillus flavus*. Moreover, the MAP kinase Fus3 (AnFus3) interacts with the conserved nuclear transcription factor AnSte12 to initiate sexual development and phosphorylates VeA, which is a major regulatory protein required for sexual development and coordinated secondary metabolite production in *A. nidulans* (Bayram et al., 2012). In *G. lucidum*, APSES transcription factor (GlSwi6) (Zhang et al., 2018; Lian et al., 2022), GATA transcription factor (AreA) (Zhu et al., 2019), Cys2His2 zinc finger protein gene C2H2-type transcription factor (CRZ1) (Li and Zhong, 2020), and Pcc1 transcription factor (PacC) (Wu et al., 2016) were found to play a role in fungal growth, fruiting body development and GA biosynthesis. We previously identified and characterized a transcription factor MADS1, which can regulate GA biosynthesis, and the gene-silencing mutants of MADS1 hinder the formation of the primordia in *G. lucidum* (Meng et al., 2021). Collectively, these studies have provided insights into molecular mechanisms and pathways that link chemical and morphological differentiation processes in fungi.

Although previous reports have shown that most secondary metabolites are produced by organisms that exhibit filamentous growth and have a relatively complex morphology, the mechanism underlying this connection unclear.

In the present study, we performed transcriptome and metabolome analyses to identify genes involved in GA biosynthesis during development of *G. lucidum*. Particularly, we evaluated the effect of transcription factors in GA biosynthesis by quantitatively analyzing GA accumulation, intermediate formation, and gene expression of key regulatory enzymes. This is the first time to investigate the global changes of transcription factors during development of *G. lucidum*. Taken together, our results provide invaluable insights into the connection between secondary metabolism and fruiting body formation in *G. lucidum*, and are expected to guide future studies.

## Materials and methods

### Fungal strains and culture conditions

A *G. lucidum* strain (accession number: ACCC53264) was provided by Prof. Mingwen Zhao of Nanjing Agricultural University, and preserved at the Agricultural Culture Collection of China. Fungal mycelia were cultured in complete yeast medium (CYM), comprising 1% maltose, 2% glucose, 0.2% yeast extract, 0.2% tryptone, 0.05% MgSO_4_ 7H_2_O, and 0.46% KH_2_PO_4_, and maintained at 28^°^C. Spawn was prepared in polypropylene bags and incubated at growth conditions described in a previous study (Meng et al., 2019). Samples were collected at three stages, namely mycelia, primordia, and fruiting body, then stored in liquid nitrogen.

### RNA sequencing and transcriptomic analysis

RNA was isolated from samples (Mycelia, Primordia, and Fruiting body), and detected RNA concentration and purity using previously described methods (Meng et al., 2022). 1.5 μg of total RNA per sample was reverse transcribed to complementary DNA (cDNA). The cDNA was purified and ligated to sequencing adapters, then resolved on an agarose gel. cDNA fragments (300 bp) were extracted from the gels, purified and enriched by PCR to construct the final cDNA library (Mycelia, Primordia, and Fruiting body). Libraries were generated using the NEBNext^®^ Ultra™ RNA Library Prep Kit for Illumina^®^ (NEB, United States). Clustering of the index-coded samples was performed on a cBot Cluster Generation System using TruSeq PE Cluster Kit v3-cBot-HS (Illumina) according to the manufacturer’s instructions. Next, libraries were sequenced on the Illumina Hiseq platform, to generate 150-bp paired-end reads. About 47 M raw reads were generated per sample.

Raw reads (fastq format) were first processed through in-house perl scripts to remove adapters, as well as low-quality reads and those containing poly-N. At the same time, we calculated Q20, Q30, GC-content and sequence duplication level of the clean data. All other downstream analyses were performed on clean, high-quality data. The transcriptome was assembled by mapping the reads to *G. lucidum* reference genome (Project accession number PRJNA71455) (Chen et al., 2012). Transcript abundances were presented as normalized fragments per kb of transcript per million mapped reads. A gene was considered to be significantly differentially expressed if its expression differed between two samples by a fold change > 2 and a *p*-value < 0.05.

Annotations, to identify gene function, were performed on the following databases: NCBI non-redundant (Nr) protein sequences; NCBI non-redundant nucleotide sequences (Nt); Protein family (Pfam); Clusters of Orthologous Groups of Proteins (KOG/COG); Swiss-Prot (A manually annotated and reviewed protein sequence database); KEGG Ortholog database (KO); and Gene Ontology (GO).

### Sample preparation and LC-MS analysis

Samples for LC-MS analysis were prepared as previously reported (Meng et al., 2022). Briefly, 80 mg of each sample was extracted by 1 mL solution of methanol-water (7: 3, v/v). Ultrasonic extraction was performed in an ice-water bath for 30 min, after which extracts were stored overnight at −20^°^C. The supernatants were then collected, filtered through 0.22 μm polyvinylidene fluoride membranes, and stored at −80^°^C until subsequent LC-MS analysis. All sample extracts were mixed with 20 μL of 2-chloro-l-phenylalanine (0.3 mg/mL methanol), as an internal standard.

To identify differentially accumulated metabolites cross various periods, we randomly analyzed 18 samples from three independent biological replicates *via* LC-MS as described previously (Meng et al., 2022). Chromatographic separation of samples was performed using an ACQUITY UPLC HSS T3 column (2.1 mm × 100 mm, 1.8 μm) equipped with a binary solvent system (solvent A: 0.1% formic acid in deionized water; solvent B: 0.1% formic acid in acetonitrile). The following gradient elution procedure was used: 0–2 min, 5% B; 2–4 min, 5% B; 4–8 min, 30% B; 8–10 min, 50% B; 10–14 min, 80% B; 14–15 min, 100% B; 15.1 min, 5% B and 16 min, 5% B. The process included a flow rate of 0.35 mL/min, an injection volume of 2 μL, and column temperature maintained at 45^°^C. Instrument settings were as follows: Ion source: ESI; capillary temperature: 320°C; spray voltages: (+3.8, -3) kV; mass scan range: 100–1200; resolution (full scan): 70000; resolution (HCD MS/MS scans): 17500; sheath gas flow rate (Arb): 35 (positive ion) and 35 (negative ion); aux. gas flow rate (Arb): 8 (positive ion) and 8 (negative ion). Metabolomics data were deposited to the EMBL-EBI MetaboLights database (Haug et al., 2019).

### Transcription factors identification

Identification of transcription factors were according to Fungal Transcription Factor Database (FTFD), which was designed for integrating the putative fungal transcription factors and its references. This site provides sequences, taxonomical and phylogomic context of all putative fungal transcription factors. Transcription factor family map and transcription factor matrix will provide overall sketch of all fungal transcription factors. FTFD included 123,899 fungal and Oomycetes transcription factors, 75,064 transcription factors belonging to phylum Ascomycota, and 37,831 transcription factors belonging to the phylum Basidiomycota, and others belonging to Chytridiomycota, Microsporidia, and Peronosporomycota. Blastp was used to find paralogs for all proteins (initial blastp parameters at limit expect value < 1e-5, and matrix was BLOSUM62).

### Metabolite identification

Identification of differentially expressed metabolites in the raw data was performed using the UNIFI 1.8.1 software. Baseline filtration, peak identification, peak alignment, peak filling, retention time (RT), and normalization of the raw data were statistically analyzed using QI v2.3 (Waters Corporation, Milford, United States). Metabolite identification was performed based on exact mass to charge ratios (m/z), isotope distributions, fragmentation patterns and database hits (The Human Metabolome Database, Lipidmaps, and METLIN). Additionally, we applied a self-written R package and in-house self-built secondary mass spectrometry database containing 550 metabolites for metabolite identification. Data processing parameters were as follows: precursor tolerance 5 ppm, fragment tolerance 10 ppm, and product ion threshold 5%. Compounds with more than 50% missing values, for each condition, were eliminated while the remaining missing values were replaced by half of the minimum value. Qualitative data were analyzed according to the score of qualitative results. Compounds with a score of more than 36 (a full score of 60) and less than 36 were accepted and deleted, respectively. The maximum total score was 60 points, allocated as follows: 20 points for MS/MS matching, 20 points for MS/MS fragmentation matching, and 20 points for isotopic distribution matching.

### Determination of total ganoderic acid and flavonoid contents

Ganoderic acid content and flavonoid contents were measured as described in our previous studies (Meng et al., 2019; Meng et al., 2021).

### Analysis of gene function

Functional analyses were performed *via* gene silencing and fungal transformation, using constructs developed as previously described (Meng et al., 2021). Briefly, the fungal RNAi vector pAN7-ura3-dual was used to silence genes encoding homeobox transcription factor and velvet family proteins in *G. lucidum*. The cDNA fragment was digested with *Kpn*I and *Spe*I (Takara) and then inserted into pAN7-ura3-dual at the corresponding restriction sites. This vector was used to transform the *G. lucidum* protoplasts by liposome transformation, with a 1:1 volumetric ratio of liposome to vector. Coincubation was performed for half an hour at 4^°^C.

### Gene expression analysis

Total RNA was extracted from all samples using the RNAiso Plus Kit (Takara, Kusatsu, Japan) according to the manufacturer’s instructions. Equal concentrations of the RNA were reverse transcribed to cDNA using the TransStart All-in-One first-strand cDNA synthesis supermix (TransGen Biotech, Beijing, China). The cDNA was then used for quantitative real time PCR (qRT-PCR) using the SYBR Green kit (Bio-Rad, Hercules, United States), performed on a LightCycler 96 SW 1.1 instrument, to analyze the transcription levels of gene-silenced strains. Primer sequences for the target genes are listed in Table 1. Expression data for genes in silenced strains were normalized against the internal reference gene 18S rRNA. The relative expression levels were calculated by comparing the cycle threshold (Ct) of each target gene with the corresponding internal reference gene using the 2^–ΔΔ*Ct*^ method (Livak and Schmittgen, 2001).

**TABLE 1:** The primers in this study.

	Forward primer (5′ to 3′)	Reverse primer (5′ to 3′)
SpeI-GL25472-RNAi	ACTGACTAGTCGGTTGTAGGTTTCGTT	ACTGGGTACCGCCCTCAAGATTCGC
RT-GL25472	CCTATTGGTGGCTATCAG	GGTTCGGGGAGGTTGG
NdeI-GL25472-pGADT7	CGAAGACATATG ATGATCGGAGGCCCTG	ACTGAATTC TCATGGCTCCTCCG
HindIII-Hmgr-pABAi	GAGTAAGCTTCTGGTAAGGCGGTGTCCC	TGCTCTCGAGCAGTAGGTGGGATGGATT
HindIII-Osc-pABAi	GAGTAAGCTTCGCTTGCTCAACAACCTC	TGCTCTCGAGGAATGTTGGTTGGGTTAA
SpeI-GL19230-RNAi	CTGACTAGTATCGCCGACCTATTGACC	CTTGGTACCTGCCAGGGAACTTCTTCG
SpeI-GL21819-RNAi	TCTACTAGTGTGGACCTGCCGTTTCTGG	GTTGGTACCCGATCATTCGAGCACCTTG
RT-GL19230	CCACTGCAAATCCATCTTCTCC	GAGGGAACAGGACAGTGGAGTTG
RT-GL21819	TCCAGCATCCTCACCG	ATGGGGCGAAGACACC
RT-HMGR	GTCATCCTCCTATGCCAAAC	GGGCGTAGTCGTAGTCCTTC
RT-SQS	CTGCTTATTCTACCTGGTGCTACG	GGCTTCACGGCGAGTTTGT
RT-OSC	AGGGAGAACCCGAAGCATT	CGTCCACAGCGTCGCATAAC
RT-18S	TATCGAGTTCTGACTGGGTTGT	ATCCGTTGCTGAAAGTTGTAT

### Yeast one-hybrid assays

Yeast one hybrid assays were conducted using the Matchmaker One-hybrid System (Clontech). Briefly, a 100-bp AATT region surrounding the *osc* and *hmgr* promoter sequences was synthesized with *Hind* III and *Xho* I flanking restriction sites, then cloned into pAbAi. Next, *GL25472* cDNA were cloned into the pGADT7 vector for expression in yeast. Plasmids for yeast one-hybrid were co-transformed into yeast Gold strain, then cells harboring the target plasmids cultivated in SD medium lacking leucine containing 200 ng/μL Aureobasidin A (AbA). The primers sequences of the target fragments used are outlined in Table 1.

## Results

### Differential gene expression profiles

**FIGURE 1:**
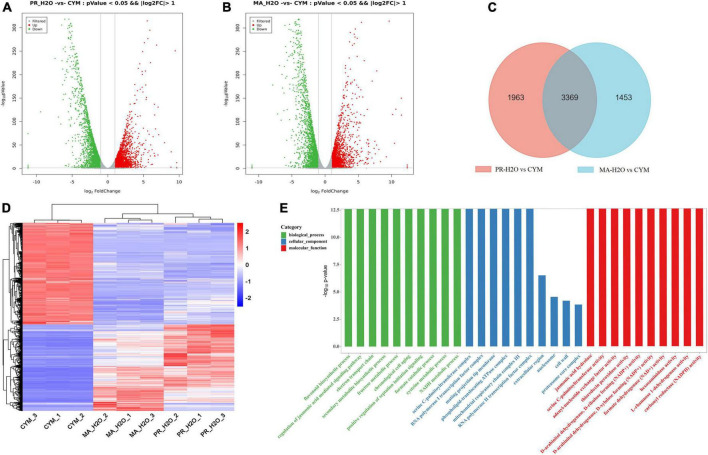
The transcriptome analysis in different developmental stages. Panels **(A,B)** are the DEGs in PR-H_2_O vs. CYM and MA-H_2_O vs. CYM, respectively. Panel **(C)** is Venn diagram of DEGs between (PR-H_2_O vs. CYM) and (MA-H_2_O vs. CYM). Panels **(D,E)** are the heat map and GO enrichment analysis of common genes. CYM, mycelia; PR-H_2_O, primordia; MA-H_2_O, fruiting body. There are three replicates in each sample of transcriptome.

We elucidated the relationship between secondary metabolites and fruiting body formation at different developmental stages using transcriptome and metabolome profiling, including mycelia (CYM), primordia (PR-H_2_O), and fruiting body (MA-H_2_O). Transcriptome data were deposited in the NCBI database, under accession numbers PRJNA825330 and PRJNA769204. Results revealed 5332 and 4822 differentially expressed genes (DEGs) between CYM and PR-H_2_O, MA-H_2_O, respectively (Figures 1A,B and Supplementary Tables 1, 2). Among them, 3369 DEGs were common between PR-H_2_O vs. CYM and MA-H_2_O vs. CYM (Figure 1C). To test the level of coregulation among differentially expressed genes, we performed cluster analysis of the 3,369 differentially regulated genes (Figure 1D), and found that most genes were highly expressed in mycelia but lowly in both primordia and fruiting body except a few genes. Interestingly, genes were differentially expressed between mycelia and primordia, but not between primordia and fruiting body. Results from GO enrichment analysis showed that 3,369 common DEGs were enriched in various terms, namely flavonoid biosynthetic process, regulation of the jasmonic acid mediated signaling pathway, electron transport chain, and secondary metabolite biosynthetic process (Figure 1E). Moreover, primordia not only represented a transition from vegetative to reproductive growth, but was also an important turning point during fungal growth and development. GO terms suggested that secondary metabolite biosynthetic process significantly changed during fungal development.

### Metabolite profiles during development of *Ganoderma lucidum*

**FIGURE 2:**
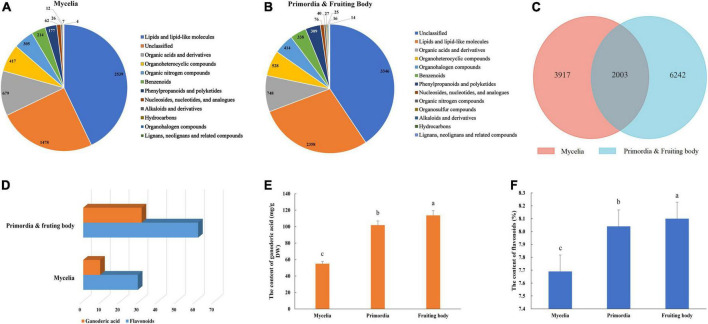
Metabolite identification in the development of *G. lucidum*. Panels **(A,B)** are the kinds of metabolites in the Mycelia, and Primordia and Fruiting body. Panel **(C)** the is Venn diagram of common metabolites in the Mycelia and Primordia and Fruiting body. Panels **(D–F)** are the kinds of secondary metabolites, contents of GAs and flavonoids. Means within a column carrying the same letter are not significantly different. Different superscript lowercase letters (a, b, and c) indicate significant differences (*p* < 0.05).

Non-targeted metabolomics analysis revealed a total of 16,327 metabolites in mycelia of *G. lucidum*, of which 5,920 were successfully annotated (Figure 2A and Supplementary Table 3). The major metabolites included lipids and lipid-like molecules, organic acids and derivatives, organoheterocyclic compounds, and organic nitrogen compounds. Notably, 24,876 metabolites were detected in the primordia and fruiting body stages, of which 8,245 metabolites were successfully annotated (Figure 2B and Supplementary Table 4). Mycelia, primordia and fruiting body stages resulted in largely similar types of metabolites, with a total of 2003 found to be common among the aforementioned stages (Figure 2C). Notably, there were 32 and 61 compounds annotated as GAs and flavonoids in the primordia and fruiting body, which were more than those obtained in mycelia (10 and 29 compounds annotated as GAs and flavonoids) (Figure 2D and Supplementary material). In addition, GA and flavonoid contents in the primordia and fruiting body were higher than those obtained in mycelia (Figures 2E,F).

### Effect of development on the expression of key ganoderic acid biosynthesis genes

**FIGURE 3:**
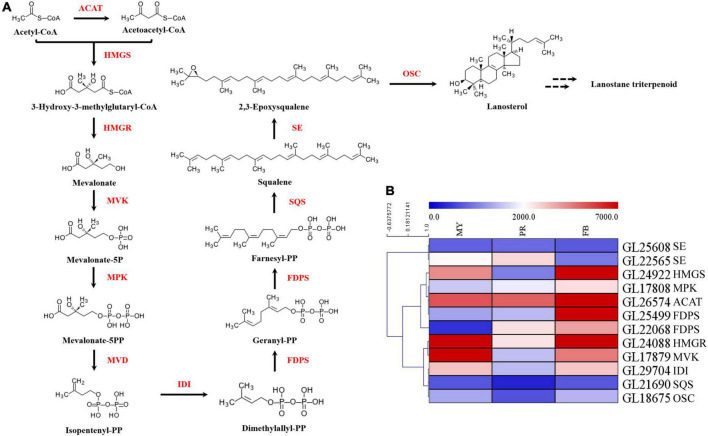
The key genes of enzymes in ganoderic acid biosynthesis pathway of *G. lucidum.* Panel **(A)** the terpenoid backbone biosynthesis in *G. lucidum*. Red represents the enzyme, and black represents the metabolites. Panel **(B)** the gene expression levels of enzymes involved in terpenoid backbone biosynthesis. ATAC, acetyl-CoA -acetyltransferase; HMGS, hydroxymethylglutaryl-CoA synthase; HMGR, 3-hydroxy-3-methylglutaryl-coenzyme A reductase; MVK, mevalonate kinase; MPK, phosphomevalonate kinase; MVD, pyrophosphomevalonate decarboxylase; IDI, isopentenyl-diphosphate isomerase; FDPS, farnesyl diphosphate synthase; SQS, squalene synthase; SE, squalene epoxidase; OSC, 2,3-oxidosqualene-lanosterol cyclase. MY, mycelia; PR, primordia; FB, fruiting body.

In order to elucidate mechanism underlying the effect of development on triterpenoid biosynthesis, we examined encoding enzymes involved in terpenoid backbone biosynthesis (Figure 3A). Results showed that phosphomevalonate kinase (MPK, *GL17808*), acetyl-CoA acetyltransferase (ACAT, *GL26574*), and farnesyl diphosphate synthase (FDPS, *GL25499*, GL22068) genes were significantly upregulated with fruiting body development (Figure 3B). Specifically, MPK, ACAT, and 2 FDPSs exhibited a 1.82-fold, 1.97-fold, 7.85-fold, and 10.60-fold upregulation in the fruiting body, respectively. Moreover, squalene monooxygenase (SE, *GL22565*) exhibited a 1.58-fold upregulation in the primordia compared with mycelia, while isopentenyl-diphosphate isomerase (IDI, *GL29704*), squalene synthase (SQS, *GL21690*) and oxidosqualene cyclase (OSC, *GL18675*) exhibited a 1. 06-, 1. 10-, and 1.16-fold upregulation, respectively, in the fruiting body relative to mycelia. Notably, the highest expression for most genes encoding enzymes was recorded during primordia or fruiting body developmental periods (Figure 3).

### Transcription factors in *Ganoderma lucidum*

Transcription factors regulate gene expression in a cell, thus in many respects, their repertoire determines the cell’s life and functionality. To better understand the regulatory mechanisms underlying fungal development and secondary metabolism, we analyzed expression patterns of transcription factors during *G. lucidum* development.

**FIGURE 4:**
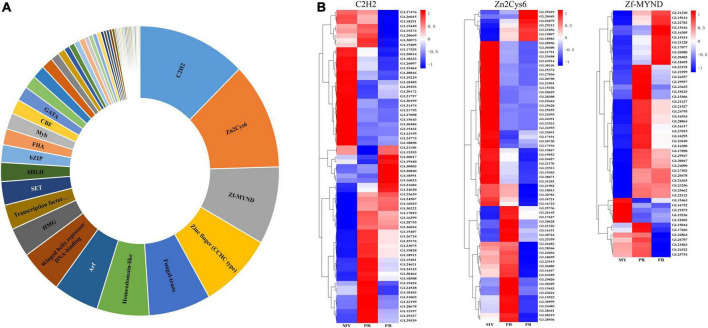
The transcription factors in *Ganoderma lucidum.* Panel **(A)** is the transcription factors family in *G. lucidum*. Panel **(B)** is the expression levels of C2H2, Zn2Cys6, and Zf-MYND in the development of *G. lucidum*. MY, mycelia; PR, primordia; FB, fruiting body.

Consequently, we identified a total of 516 homologous protein of transcription factors in *G. lucidum*. Among them, 90 had more than two domains, and belonged to 53 transcription factor families (Figure 4A and Supplementary Table 5). The highest number of significantly affected genes encoding transcription factors belonged to the C2H2 transcription factor family, followed by Zn2Cys6 and Zf-MYND (Figure 4B). A total of 110 differentially expressed transcription factors were recorded across the three stages (Supplementary Table 6). Moreover, 67 C2H2, 70 Zn2Cys6, and 50 Zine finger-MYND (Zf-MYND) transcription factors, including 24 DEGs of C2H2, 27 DEGs of Zn2Cys6, and 13 DEGs of Zf-MYND were expressed across the three stages Interestingly, mycelia exhibited the highest transcriptional levels for 15 DEGs of C2H2 (62.5%), and 24 DEGs of Zn2Cys6 (88.9%) transcription factors, while other DEGs expressed in the primordia or fruiting body. The primordia or fruiting body exhibited the highest transcriptional levels for 10 DEGs of Zf-MYND (76.9%) transcription factors. Collectively, these results suggested that these transcription factor homologs might be vital regulators of gene expression and play different roles at different developmental stages of *G. lucidum*.

### Homeobox transcription factor regulates the ganoderic acid biosynthesis

**FIGURE 5:**
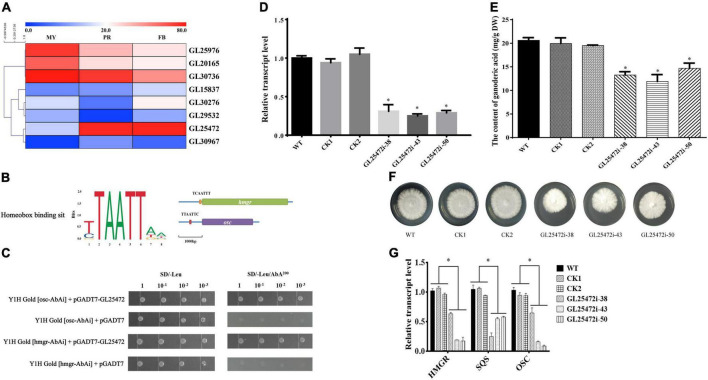
The effects of homeobox transcription factors on ganoderic acid biosynthesis in *G. lucidum.* Panel **(A)** is the gene expression level of homeobox transcription factors. MY, mycelia; PR, primordia; FB, fruiting body. Panel **(B)** is the binding sit of homeobox transcription factor. The orange box (TCAATTT) and red box (TTAATTC) were CREs located in the promoter region of hmgr and osc. Panel **(C)** is yeast one-hybrid assay. Panels **(D,E)** are the relative transcript levels and GA contents of gene-silencing strains. Panel **(F)** the mycelial colony. Panel **(G)** the transcript levels of the genes encoding key enzymes. WT (wild-type), CK1 and CK2 (the empty vector controls), *GL25472* (38, 43, 50) were gene-silencing strains. HMGR, 3-hydroxy-3-methylglutaryl-coenzyme A reductase; SQS, squalene synthase; OSC, 2,3-oxidosqualene-lanosterol cyclase. The histograms marked with * represent significant differences; it indicates statistical significance (*p* < 0.05) compared to WT.

Although homeobox transcription factors have been implicated in secondary metabolism in fungi (Cary et al., 2017), their roles in GA biosynthesis in *G. lucidum* are largely unknown. To further identify the relationship between homeobox transcription factors and GA biosynthesis, we annotated 9 homeobox transcription factors from the fungal transcription factor database, including 1 gene (*GL30604*) that was not expressed (Figure 5A). To screen the key homeobox transcription factors, we analyzed 1 kb promoter sequences upstream of *hmgr* and *osc* (pHmgr and pOsc) coding region *via* Yeastract database, and compared the *cis-*regulatory elements (CREs) present in pHmgr and pOsc with homeobox protein YOX1 in *S. cerevisiae*. Previous studies have shown that many common CREs in corresponding sites shared by pHmgr and pOsc can be recognized by many transcriptional factors (Guo et al., 2015). In the present study, we found that a homeobox transcription factor GL25472 contained CREs located in the promoter region of *hmgr* and *osc* (Figure 5B), and Yeast-one-hybrid results showed that *GL25472* could bind to the promoter regions of *hmgr* and *osc* (Figure 5C). Therefore, we selected *GL25472* for further investigations.

To determine whether *GL25472* influences GA biosynthesis in *G. lucidum*, we constructed an RNAi vector targeting the *GL25472* gene. qRT-PCR results revealed silencing efficiencies of 70, 75, and 71% for *GL25472i-38*, *GL25472i-43*, and *GL25472i-50*, respectively (Figure 5D). Moreover, *GL25472i-38*, *GL25472i-43*, and *GL25472i-50* silencing constructs mediated a 35.73, 42.33, and 28.91% reduction in GA content of mycelia, respectively, compared to the WT strain (Figure 5E). Strains in which the *GL25472* had been silenced also exhibited reduced growth rates (Figure 5F). Moreover, silencing of the *GL26472* mediated a significant downregulation of *HMGR*, *SQS*, and *OSC*, key genes involved in GA biosynthesis, relative to WT strain (Figure 5G).

### Velvet family regulates the ganoderic acid biosynthesis

**FIGURE 6:**
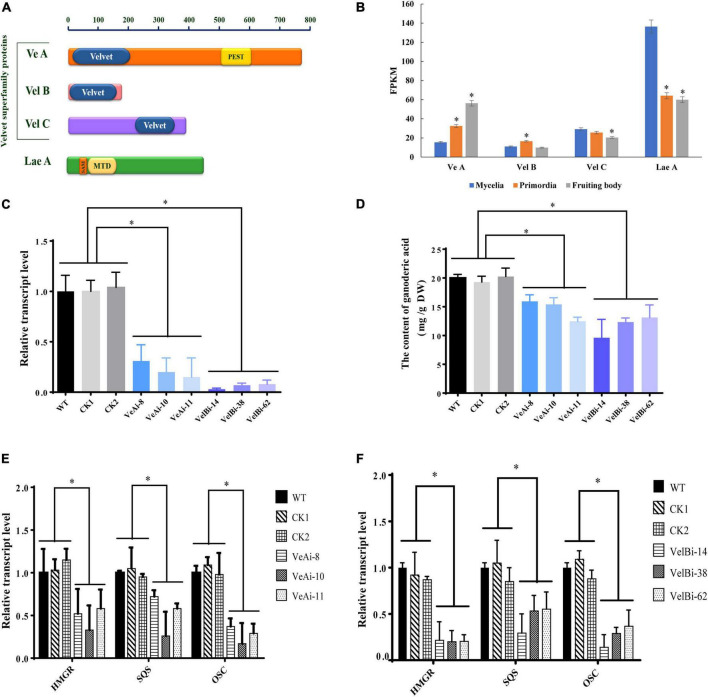
The effects of velvet family on ganoderic acid biosynthesis in *G. lucidum.* Panel **(A)** is predicted conserved domains. Panel **(B)** the gene expression level in development of *G. lucidum*. FPKM, Fragments Per Kilobase Million. Panels **(C,D)** are the gene expression levels and GA contents of VeA and VelB. Panels **(E,F)** are the transcript levels of the genes encoding key enzymes. WT (wild-type), CK1 and CK2 (the empty vector controls), VeAi (8, 10, 11) and VelBi (14, 38, 62) were gene-silencing strains. HMGR, 3-hydroxy-3-methylglutaryl-coenzyme A reductase; SQS, squalene synthase; OSC, 2,3-oxidosqualene-lanosterol cyclase. The histograms marked with * represent significant differences; it indicates statistical significance (*p* < 0.05) compared to WT.

Previous studies have shown that the VeA-VelB-LaeA complex can activate secondary metabolism in fungi (Bayram et al., 2008). Therefore, we analyzed expression levels of members of the velvet family and LaeA across different development stages. We annotated three genes encoding velvet family protein, including VeA (*GL19230*), VelB (*GL21819*), and VelC (*GL23472*), and one gene (*GL26103*) encoding LaeA (Figure 6A). All velvet family proteins contain a velvet conserved domain, while VeA has a proline glutamic acid serine and threonine (PEST) rich sequence. Additionally, LaeA contains a S-adenosyl methionine-binding site (SAM) and a methyltransferase domain (MTD) in its amino acid sequence. Notably, VeA expression was upregulated during fruiting body development, with the highest level recorded at the fruiting body stage (Figure 6B). By contrast, VelB showed relatively high expression at primordia stage, while VelC was lowly expressed at the fruiting body stage. LaeA was down-regulated at both primordia and fruiting body stages.

To further study the function of velvet family proteins, we separately silenced two important key velvet family proteins, namely VeA and VelB, in *G. lucidum via* RNAi (Figure 6C). qRT-PCR results revealed a silencing efficiency of > 70%. Results showed that silencing of *VeA* and *VelB* mediated a significant reduction in GA content of mycelia compared to the WT strain (Figure 6D). Moreover, *HMGR*, *SQS*, and *OSC*, key genes involved in GA biosynthesis, were markedly down-regulated in *VeA* and *VelB* silencing strains compared to WT (Figures 6E,F). Collectively, these results indicated that velvet family proteins play a key role in development and GA biosynthesis in *G. lucidum*.

## Discussion

Transcription factors play essential roles in the signal transduction pathway, being the last link between signal flow and target genes expression. Fungi have significantly lower number of experimentally verified transcription factors compared to higher eukaryotes, as reflected in databases such as TRANSFAC (Wingender et al., 1996) and MycoPath. This phenomenon may be attributed to two possible scenarios: either they are really less abundant or they have not yet been identified. In this study, we estimated the potential mechanism underlying regulation of secondary metabolism during development of *G. lucidum*.

The transcriptional regulation process is a complex and dynamic process that is regulated by transcription factors. Expression of developmental transcription factors has also been found to be transiently present. Therefore, we quantified gene expression using previously described datasets (Chen et al., 2012). Although most gene expression models were similar in available transcriptome data, there were also localized distinctions between transcriptome data from different strains and growth status. In this study, transcriptome data revealed differentially expressed genes between mycelia and primordia, as well as between mycelia and the fruiting body. The difference is likely caused by two reasons. On the one hand, the discrepancy of gene expression profile could be due to differences *Ganoderma* in important aspects of growth and development. On the other hand, it could also be due to the differences between media.

Moreover, metabolome is often thought of as a “readout” of physiological states. Therefore, it is widely used in the study of the growth and development of organisms. Notably, mycelia, primordia and fruiting body stages resulted in largely similar types of metabolites, the major metabolites included lipids and lipid-like molecules, organic acids and derivatives, organoheterocyclic compounds, and organic nitrogen compounds. However, a large diversity of metabolites between mycelia and primordia/fruiting body were also significantly different. There were 3,917 specific metabolites in mycelia, and 6,242 specific metabolites in primordia and fruiting body stages. Previous report has revealed that fungal lipids are involved in organism development as well as regulatory machinery for secondary metabolism (Tugizimana et al., 2019). In this regard, linolenic -, oleic -, nonanoic - and decanoic acids have been shown to participate in the regulation of spore development and mycotoxin production in *Aspergillus* spp., and *Fusarium verticillioides* (Christensen and Kolomiets, 2011). Concerning secondary metabolites during developmental stages, ten out of the thirty-two GAs in the primordia/fruiting body stages were same with those in mycelia stage. Twenty-two GAs were unique in primordia/fruiting body, such as Ganoderenic acid B, Ganoderenic acid A, and so on, and the flavonoids greatly varied between mycelia and primordia/fruiting body (Supplementary material). These results implied that secondary metabolite patterns were altered as development progresses. The mechanism for this association may be multifaceted. One speculation might be perhaps that the variation in development resulted in diversity of metabolites. Another speculation might be perhaps that the various metabolites had an impact on the fungal development.

**FIGURE 7:**
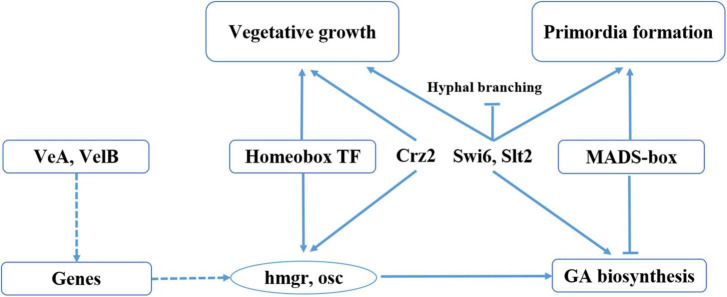
Model of regulation of fruiting body development and GA biosynthesis in *G. lucidum*. Solid lines represent the determined regulatory effect, dotted lines represent the regulatory effect of an unknown regulatory mechanism, arrows represent the facilitated effect, and short lines represent inhibition.

This is the first time a direct link has been shown between fruiting body development and GA biosynthesis in *G. lucidum* (Figure 7). In this study, we found that the homeobox transcription factor and velvet family protein played an important role in regulating the GA biosynthesis in during *G. lucidum* development. We previously identified and characterized a transcription factor *MADS1*, which can regulate GA biosynthesis, and the gene-silencing mutants of *MADS1* hinder the formation of the primordia in *G. lucidum* (Meng et al., 2021). Previous studies have shown that strains in which the Cys2His2 zinc finger protein gene C2H2 has been inactivated are arrested at the aggregate stage (Ohm et al., 2011). Results of the present study revealed that C2H2 transcription factors were upregulated in mycelia than in both primordia and fruiting body, a phenomenon that may contribute to mycelia aggregation. Studies have also shown that C2H2 transcription factors, such as *glcrz2*, play an important role in secondary metabolism (Li and Zhong, 2020). Notably, knocking out *glcrz2* resulted in marked attenuation of both mycelia growth and GA synthesis, and this was accompanied by a decline in GA production even with Ca^2+^ treatment. In addition, Zhang et al. (2018) found that silencing *GlSwi6* reduced fungal growth and increased hyphal branching, and the *GlSwi6*-silenced strains did not exhibit primordium or fruiting body formation. Moreover, the GA level of the *GlSwi6*-silenced strains decreased approximately 25% compared with those of the WT strain. The similar phenotypic characteristics were found in knockdown of *GlSlt2* (Zhang et al., 2017b). These results showed that *GlSwi6* and *GlSlt2* were involved in fungal growth, development and GA biosynthesis in *G. lucidum*.

Furthermore, there are several no defined InterPro Term in the Fungal Transcription Factor Database, such as the heat shock factor. Previous studies have demonstrated that heat stress modulates heat shock protein expression, thereby affecting GA biosynthesis and hyphal branching of *G. lucidum via* Cytosolic Ca^2+^. However, the identified transcription factors might be less than the actual number in *G. lucidum*, due to lack the defined InterPro. The large array of transcription factors annotated in the present study warrant further research attention. There is also need to investigate many aspects, such as the function of transcription factors, gene interactions, and transcriptional regulatory networks, among others.

## Conclusion

In summary, transcription factors act as an important link between secondary metabolites biosynthesis and fungal development. In the present study, we found that genes were differentially expressed between mycelia and primordia. GO terms of the DEGs revealed that secondary metabolite biosynthetic process significantly changed during fungal development. A set of 53 potential transcription factor families was annotated in *G. lucidum*. Notably, we found homeobox transcription factor and velvet family protein played important role in GA biosynthesis. Combined with previous studies, we provided a model diagram of transcription factors involved in GA biosynthesis during fruiting body formation. Taken together, these findings are expected to significantly improve our understanding of secondary metabolites biosynthesis and development in fungi.

## Data availability statement

The datasets presented in this study can be found in online repositories. The names of the repository/repositories and accession number(s) can be found in the article/Supplementary material.

## Author contributions

LM: conceptualization, funding acquisition, and writing – original draft. RZ: methodology. XZ: investigation. JL: formal analysis. QW: visualization. PW: software. LW: project administration. ZL: supervision. WW: writing – review and editing. All authors contributed to the article and approved the submitted version.
